# Quantum Dot-Hybridized Temperature- and Salt-Resistant Polyacrylamide for Enhanced Oil Recovery in High-Temperature and High-Salinity Reservoirs

**DOI:** 10.3390/polym18172084

**Published:** 2026-08-28

**Authors:** Hua Li, Jingjing He, Rui Jing, Song Wang, Aihui Li, Ting Chen, Daijun Du, Suhan Zhang

**Affiliations:** 1 Xi’an Changqing Chemical Group Co., Ltd., Xi’an 710200, China; lhua28_cq@petrochina.com.cn (H.L.);; 2 Engineering Technology Department, PetroChina Xinjiang Oilfield Company, Karamay 834000, China; 3 State Key Laboratory of Oil and Gas Reservoir Geology and Exploitation, Southwest Petroleum University, Chengdu 610500, China; 4 School of Petroleum and Natural Gas Engineering, Chongqing University of Science and Technology, Chongqing 401331, China

**Keywords:** quantum dot-hybridized polymer, temperature and salt resistance, polymer flooding, rheological properties, EOR

## Abstract

Conventional partially hydrolyzed polyacrylamide (HPAM) suffers severe chain coiling, viscosity attenuation and precipitation under high-temperature and high-salinity reservoir brines, restricting its tertiary oil recovery efficiency. Herein, a novel carbon quantum dot hybrid terpolymer (QDHSTP) was synthesized via free-radical copolymerization, where silane-modified nitrogen-doped carbon quantum dots (FNCQDs) were covalently bonded to acrylamide (AM)/2-acrylamido-2-methylpropane sulfonic acid (AMPS)/diallyldimethylammonium chloride (DMDAAC) backbones. FTIR, ^1^H NMR and thermogravimetric analysis (TGA) verified successful grafting of FNCQDs, while SEM revealed a continuous three-dimensional entangled network constructed by polymer chains. Steady and oscillatory rheology systematically characterized the solution viscoelasticity: QDHSTP solutions followed the power-law shear-thinning model, with flow behavior index *n* decreasing from 0.698 to 0.676 and consistency factor *k* rising from 80.91 to 131.13 mPa·s^n^ as concentration increased from 2000 to 3000 mg/L. All samples behaved as viscosity-dominated viscoelastic fluids, with elastic modulus exhibiting stronger frequency dependence. Benefiting from embedded FNCQDs, QDHSTP retained 79.74% and 76.06% of initial viscosity in 1.0 × 10^4^ mg/L NaCl and CaCl_2_ brine, respectively, markedly better than that of the polymer without incorporated FNCQDs respectively, and maintained thickening capacity at 90 °C. Artificial sandstone core flooding demonstrated an incremental oil recovery of 29.8% over baseline waterflooding, attributed to mobility control and elastic residual oil stripping. This covalent nanohybrid strategy provides a facile route to construct thermo-salt tolerant polyacrylamides, offering a promising candidate polymer for harsh oil reservoir chemical flooding.

## 1. Introduction

As one of the most mature enhanced oil recovery (EOR) techniques, polymer flooding has been widely applied in oilfields around the world for over 50 years [1]. Polymer flooding involves the addition of water-soluble polymers to the water injection system to modify the rheological properties of the displacing fluid, thereby increasing sweep efficiency and improving oil recovery [2]. Among waterflooding-based EOR projects worldwide, polymer flooding accounts for approximately 77%, while polymer-surfactant flooding accounts for about 23% [3]. As a key component of chemical flooding, polymer flooding originated in the United States in the 1950s, with the first field pilot test conducted in 1964. Polymer flooding can generally increase oil recovery by 5% to 20% [4]. In 2006, the technology achieved a successful application at the Pelican Lake field in Canada, where oil production increased from 43 bbl/d to over 700 bbl/d and remained at a high level for more than six years [5]. In the Dina Cretaceous field in the Upper Magdalena Valley Basin, Colombia, the application of crosslinked polymer flooding resulted in a significant reduction in water cut and a marked increase in oil production. For example, Well DK-24 saw its production rate rise from 80 bbl/d to 200 bbl/d [6]. In the 1960s, China initiated preliminary research on polymer flooding technology. Although the country started later than other nations in this regard, it has progressed rapidly. Among Chinese oilfields, the Daqing Oilfield has the most extensive application of polymer flooding, with cumulative crude oil production from polymer flooding exceeding 10 million tons and an incremental oil recovery factor of 10–15% [7].

Polymer flooding is a technical measure for enhanced oil recovery achieved primarily by injecting high-molecular-weight polyacrylamide into the reservoir. The EOR mechanism of partially hydrolyzed polyacrylamide mainly involves the active amide groups on its side chains, which reduce the mobility ratio of the displacing phase, increase the viscosity of both the oil and water phases, and enhance the sweep efficiency of the displacement, thereby improving oil recovery [8,9,10]. The mechanisms of polymer flooding for enhanced oil recovery can be summarized as follows: (1) The mobility ratio improvement effect of polymers reduces the mobility ratio of the water phase. (2) Viscous effect. (3) Adsorption or entrapment effect. Taken together, the above-mentioned three effects jointly contribute to the improvement of sweep efficiency and displacement efficiency [11,12,13,14,15].

Polymer flooding, as a key technology for enhanced oil recovery, has been widely applied in major oilfields [16]. However, with increasing understanding of HPAM, researchers have found that HPAM also exhibits numerous problems in practical applications. On the one hand, it suffers from poor mechanical shear stability and is prone to degradation upon prolonged storage; the carboxyl groups on the HPAM molecular chain can flocculate with divalent metal cations such as Ca^2+^ and Mg^2+^, leading to phase separation and precipitation. On the other hand, the structure and performance of HPAM are susceptible to high-temperature environments. Elevated temperatures reduce the molecular chain dimensions of HPAM, disrupt the entanglements between polymer molecules, and weaken the hydrogen bonding between HPAM and water molecules. This results in reduced hydration of HPAM, decreased resistance to polymer motion, and consequently a decline in system viscosity [17,18]. Therefore, as the difficulty of oil extraction increases and both reservoir temperature and formation water salinity continue to rise, the viscosity of polymer solutions drops significantly, thereby reducing the efficiency of tertiary oil recovery. It is evident that improving the temperature and salt resistance of polymers is one of the key issues that urgently needs to be addressed in polymer flooding technology [19,20].

Nanomaterials, owing to their exceptional properties, have been widely applied in various fields such as electronics, chemical engineering, batteries, biology, and medicine. In recent years, the rise of nanotechnology has provided new insights for addressing the aforementioned bottlenecks, and nanomaterials have gradually been employed to modify the performance of oilfield polymers [21,22]. Through physical adsorption, inorganic nanomaterials with strong thermal stability and large specific surface area, such as SiO_2_, modified SiO_2_, and clay, are introduced into the polymer matrix, significantly improving the properties of polymer solution, with viscosity retention of ~60% at 1.0 × 10^4^ mg/L NaCl and 70 °C for SiO_2_, and viscosity retention of ~55% under similar saline conditions for clay [2,21,22]. Interactions such as hydrogen bonding and electrostatic forces exist between nanoparticles and polymer molecules. The introduction of nanoparticles can enhance the rigidity of polymer molecules, inhibit polymer hydrolysis, and protect the polymer backbone. For polymer molecules, nanoparticles can act as physical crosslinking points, reinforcing intermolecular interactions and promoting the formation of a robust three-dimensional network structure. Through chemical modification, chemical reactions are utilized to form chemical bonds between nanoparticles and polymers, achieving tight combination between the two. This bonding approach can alter the surface properties and functionalities of both nanoparticles and polymers, enabling them to play a more effective role in enhanced oil recovery under reservoir conditions. Polymers also serve as stabilizers for nanoparticle dispersion systems; the introduction of polymers can inhibit nanoparticle aggregation and improve the temperature and salt resistance of the nanoparticulate dispersion system. It is evident that utilizing nanomaterials to interact with polymer matrices through electrostatic adsorption or chemical modification to generate novel effects can achieve complementary advantages between the two, and this represents a promising direction for current research. In reviewing the existing research on temperature- and salt-resistant polymers, we find that the molecular structures of conventional synthetic polymers are becoming increasingly complex. This leads to difficulties in polymer preparation, high costs, limited molecular weight growth, and challenges in controlling the performance outcomes. Moreover, the role of nanoparticles in enhancing the temperature and salt resistance of polymer systems remains insufficiently clarified, and further studies on interfacial properties, wettability, and the oil displacement process have yet to be conducted [23,24,25,26,27].

In view of the drawbacks of HPAM commonly used in oilfield polymer flooding, such as susceptibility to precipitation or mechanical degradation under high-temperature and high-salinity conditions, which significantly reduce the viscosity and other performance properties of HPAM and severely impair enhanced oil recovery efficiency, nanomaterials offer broad application prospects in tertiary oil recovery due to their good water solubility, high specific surface area, ability to reduce interfacial tension, and capacity to markedly alter rock wettability. Therefore, in this study, we report a novel quantum dot-hybridized temperature- and salt-resistant terpolymer (QDHSTP), synthesized via thermally initiated free-radical copolymerization, wherein silane-modified nitrogen-doped carbon quantum dots (FNCQDs) are covalently incorporated into the AM/AMPS/DMDAAC backbone. The specific contributions of this work are threefold: (i) we demonstrate a covalent grafting strategy that fundamentally overcomes the stability limitations of physically blended nanoparticle-polymer systems; (ii) we systematically elucidate the triple synergistic mechanisms (electrostatic shielding, preferential metal-ion coordination, and steric hindrance) by which FNCQDs confer exceptional salt and temperature resistance; and (iii) we validate the enhanced oil recovery performance of QDHSTP through core flooding experiments under simulated high-temperature (90 °C) and high-salinity (1.0 × 10^4^ mg/L) reservoir conditions, achieving 29.8% incremental oil recovery.

## 2. Experimental Section

### 2.1. Materials

Urea, sodium citrate, anhydrous ethanol, 3-Methacryloxypropyltrimethoxysilane (γ-MPS), acrylamide (AM), 2-Acrylamido-2-methylpropane sulfonic acid (AMPS), diallyldimethylammonium chloride (DMDAAC), ammonium persulfate, sodium hydroxide and sodium bisulfite were of analytical grade, purchased from Kelong Chemical Reagent Factory (Chengdu, China). Water was double-deionized with a Millipore Milli-Q system to produce the 18 MΩ deionized water. Crude oil was sourced from Changqing Oilfield (Xi’an, China), with viscosity of 2.87 mPa·s at reservoir temperature (70 °C).

### 2.2. Preparation of Quantum Dot-Hybridized Temperature- and Salt-Resistant Polymer (QDHSTP)

**Figure 1:**
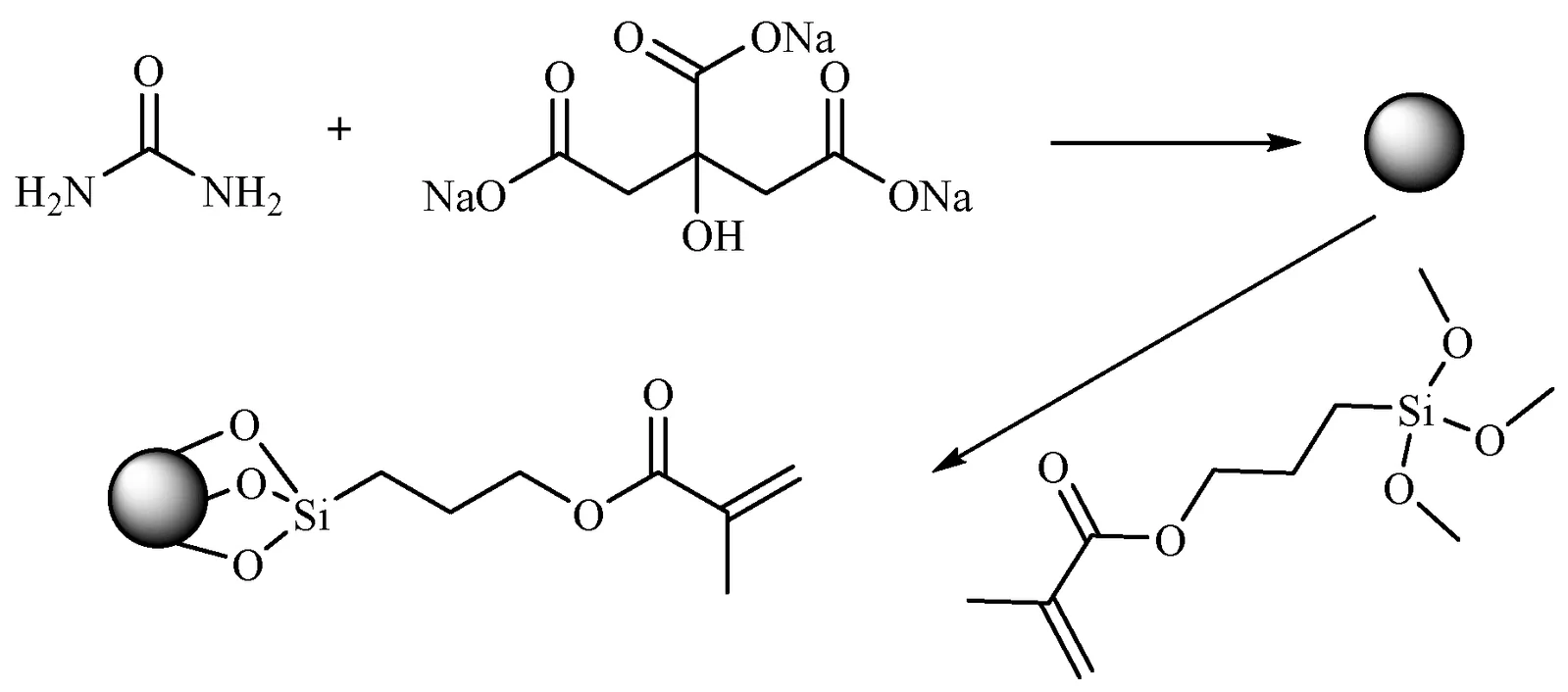
Schematic illustration of the synthetic route for functionalized quantum dots.

*Synthesis of Functionalized Quantum Dots*. Urea and sodium citrate were used as raw materials at a molar ratio of 6:1, and were ground and mixed thoroughly. The mixture was placed in an open crucible and subjected to heat treatment at 180 °C for 1 h to obtain the crude product. The crude product was washed several times with absolute ethanol under ultrasonication, dried at 70 °C, and then dispersed in ultrapure water. The dispersion was centrifuged at 4000 r/min for 10 min, and the supernatant was sequentially filtered through 0.45 μm and 0.22 μm membrane filters. The filtrate was then dialyzed using a dialysis bag with a molecular weight cutoff of 1000 Da until the external solution became colorless and clear. After drying, yellowish-brown nitrogen-doped carbon quantum dots (NCQDs) were obtained. The as-prepared NCQDs, a silane coupling agent (γ-MPS), and absolute ethanol were mixed in a three-necked flask. The reaction was carried out at 60 °C under sealed conditions with stirring at 600 r/min for 6 h. The solvent was then removed by rotary evaporation to obtain γ-MPS-modified nitrogen-doped carbon quantum dots (FNCQDs). The synthetic route is shown in Figure 1.

**Figure 2:**
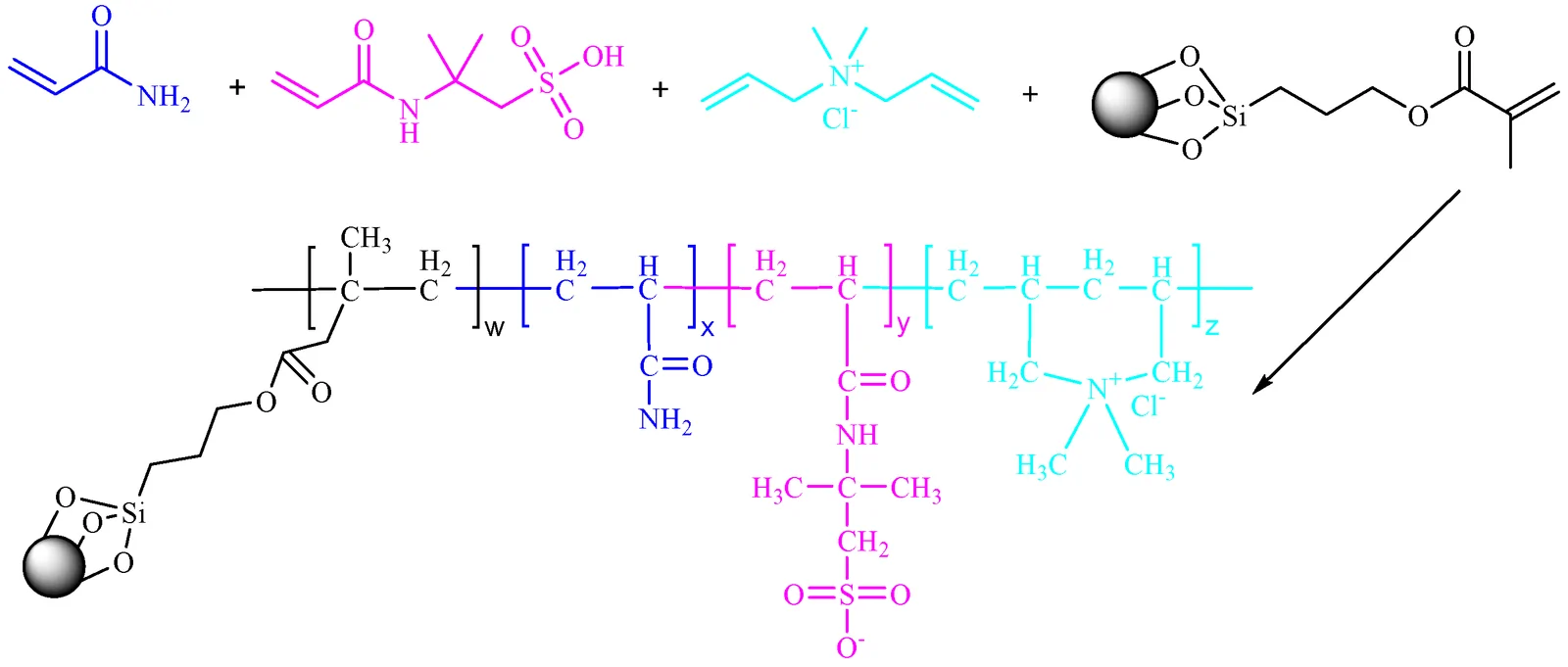
Schematic illustration of the synthetic route for QDHSTP.

*Synthesis of QDHSTP*. QDHSTP was synthesized via thermally water free-radical micellar copolymerization. 10.0 g of AM, 23.3 g of AMPS, 4.5 g of DMDAAC, and 0.1 g of FNCQDs were dissolved in 60.0 g of deionized water. Sodium hydroxide solution was used to control the pH value of the solution about 9. The dosage of ammonium persulfate and sodium bisulfite (molar ratio, 1:1) was a constant 0.2 wt% of total monomer and the percent composition of total monomer in water was 30%. Then the solution was heated to 40 °C and maintained for 4 h. QDHSTP was washed and extracted with ethanol to remove water, residual monomers and initiator. Finally, QDHSTP was further dried in a vacuous environment at 60 °C for 24 h. The synthesis routes are shown in Figure 2. To elucidate the specific contribution of FNCQDs to the polymer properties, the AM/AMPS/DMDAAC terpolymer (without FNCQDs) was synthesized under the same experimental conditions and was named HSTP for comparative purposes.

### 2.3. Characterization

The FT-IR of FNCQDs and QDHSTP were investigated using a Nicolet Nexus IR spectrometer (Thermo Fisher Scientific, Waltham, MA, USA) on KBr in the optical range of 400–4000 cm^−1^. The microstructure of FNCQDs was characterized by TEM observation, about 5 μL of FNCQDs dispersion solution was firstly added on the TEM copper grid. Then, the system was promptly immersed into a liquid ethane reservoir to be cooled by N_2_ at −160 °C. No heavy-metal staining treatment was carried out for TEM samples in this work because of FNCQDs have intrinsic electron-scattering capacity to provide sufficient contrast. The particle size distribution of FNCQDs was measured by a Mastersizer APA2000 laser particle analyzer (LPSA, Malvern, UK). The ^1^H NMR spectroscopy of QDHSTP was carried out using a Bruker A400 nuclear magnetic resonance spectrometer (Bruker Corporation, Billerica, MA, USA); the aggregating morphology of QDHSTP in deionized water and was observed by ESM (Hillsboro, OR, USA). The thermal degradation behavior of QDHSTP was investigated using a simultaneous thermal analyzer (STA 449F3, NETZSCH-Gerätebau GmbH, Selb, Germany). A temperature sweep from 40 °C to 600 °C was performed at a constant heating rate of 10 °C/min, while a continuous argon flow at 60 mL/min was maintained as the purge gas throughout the measurement.

### 2.4. Water Solubility Test

The water solubility of the polymer was assessed by monitoring the conductivity of its aqueous solution using a DDS-11A conductivity meter (Leici, Shanghai, China). A polymer sample was dissolved in deionized water at ambient temperature (approximately 25 °C). The conductivity variation was recorded over time, starting from the moment of polymer addition until a stable conductivity value was reached, and the resulting profile was used to evaluate the dissolution behavior of the polymer [1].

### 2.5. Performance Evaluation of QDHSTP Solution

As one of the most prevalent polymers applied in petroleum reservoir polymer flooding, HPAM has received considerable attention. In this work, the resistance of QDHSTP to salinity, elevated temperature, and mechanical shear was systematically investigated. Viscosity measurements were performed at 25 °C using a Brookfield DV-III rotational rheometer (Brookfield, Middleboro, MA, USA), employing spindles of varying dimensions commensurate with the viscosity of the test solutions. Rheological behavior under shear was further examined with a Haake RS600 stress-controlled rheometer (Thermo Fisher Scientific, Karlsruhe, Germany), using a cone-and-plate fixture (1° cone angle, 35 mm diameter).

### 2.6. Enhanced Oil Recovery Experiments

Artificial sandstone cores were employed as the porous medium for polymer flooding tests at a constant temperature of 90 °C, with the aim of evaluating the oil displacement performance of QDHSTP during tertiary recovery. Core characterization was undertaken to determine the baseline petrophysical parameters. The flow control efficiency of the polymer solution was quantified using the resistance factor and residual resistance factor [28,29].

(1) fr=KwμwKpμp

(2) $$f_{rr}=\frac{K_{wb}}{K_{wa}}$$

In these equations, the subscripts w and p denote the water and polymer phases, respectively. Specifically, Kw and Kp are the phase permeabilities (mD), while μw and μp are the phase viscosities (mPa·s). The additional subscripts b and a indicate measurements taken before and after polymer flooding, respectively, for the water-phase permeability (K_wb_ and K_wa_). The polymer flooding-induced increment in oil recovery, i.e., the EOR factor, was derived from the following equation:

*EOR* = *E_t_* − *E_w_* (3)

In the above equation, *E_t_* is defined as the total recovery factor for the entire flooding procedure (%), whereas *E_w_* is the recovery factor attributed to the waterflooding stage carried out before the polymer flood (%).

The displacement experiments on artificial sandstone cores were carried out following a multi-step procedure. Initially, the core properties were characterized, with the fundamental parameters compiled in Table 1. The core was then installed in the core-holder and subjected to a confining pressure at 90 °C. Following brine saturation to establish the initial water saturation (with ionic composition detailed in Table 2), oil displacement was performed using Changqing crude oil until water production ceased and the water cut was reduced to below 1%. Thereafter, the core was waterflooded to a water cut of 98%, followed by injection of a 0.3 PV polymer solution. The flooding sequence was completed with a subsequent waterflood stage, continued until the water cut reached 98% again. A fixed injection rate of 0.5 mL/min was employed for all flooding runs.

## 3. Results and Discussion

### 3.1. Characterization

**Figure 3:**
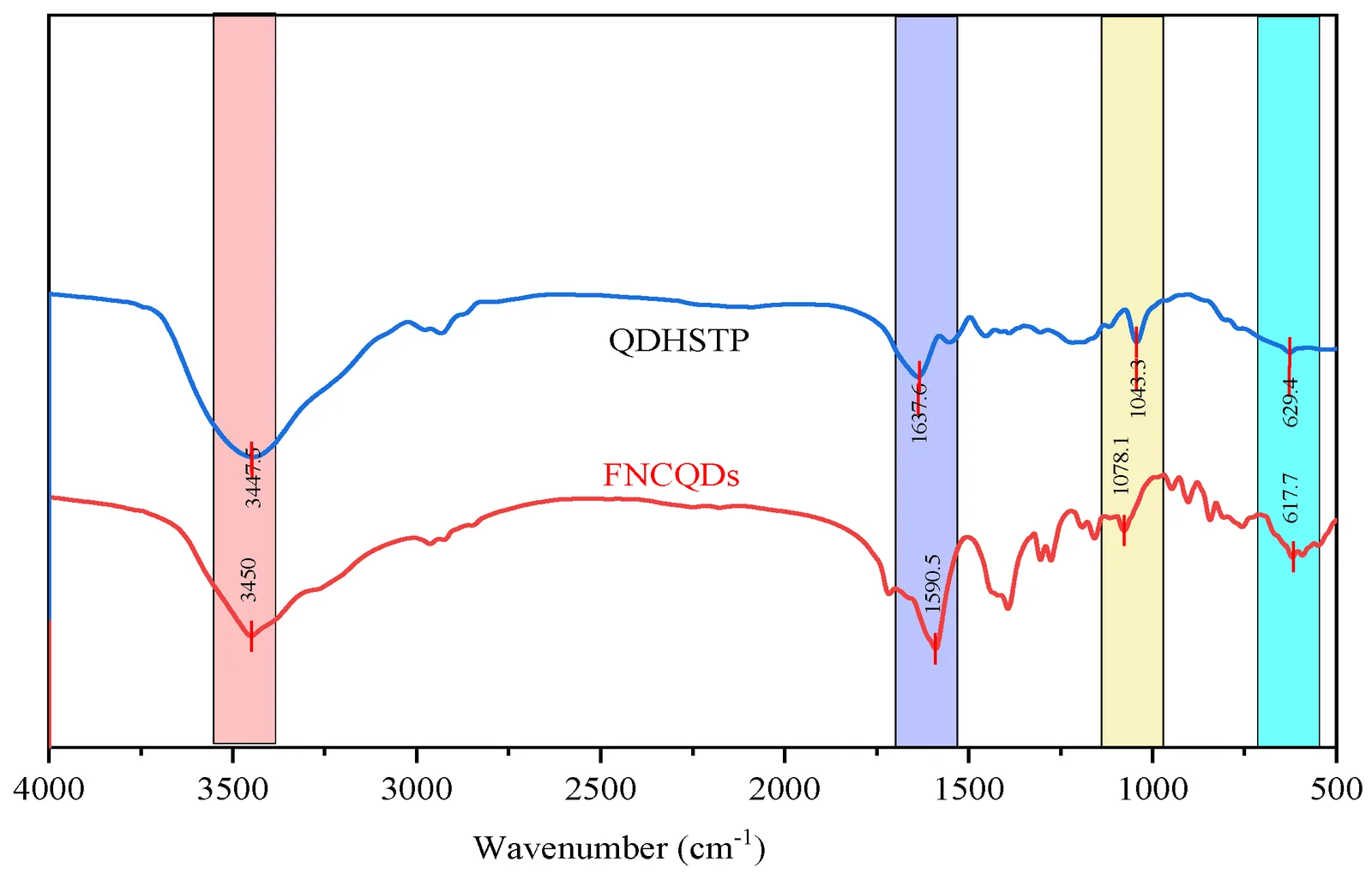
FT-IR spectra of FNCQs and QDHSTP.

The FTIR spectra of both the FNCQs and QDHSTP are illustrated in Figure 3. For the FNCQs, distinct absorption bands were identified at 3450 cm^−1^ (attributable to N-H/O-H stretching), 1590.5 cm^−1^ (N-H/C=C stretching), 1078.1 cm^−1^ (Si-O-Si/C-O-C stretching), and 617.7 cm^−1^ (NH_2_ stretching). The QDHSTP, on the other hand, displayed absorption features at 3447.5 cm^−1^ (N-H/O-H stretching), 1637 cm^−1^ (C=O/NH_2_ stretching), 1043.3 cm^−1^ (C-N/C-O-C stretching), and 629.4 cm^−1^, the latter being characteristic of the -SO_3_H moiety in AMPS. These spectral findings provide evidence of the successful fabrication of the FNCQs and their effective incorporation into the QDHSTP backbone.

**Figure 4:**
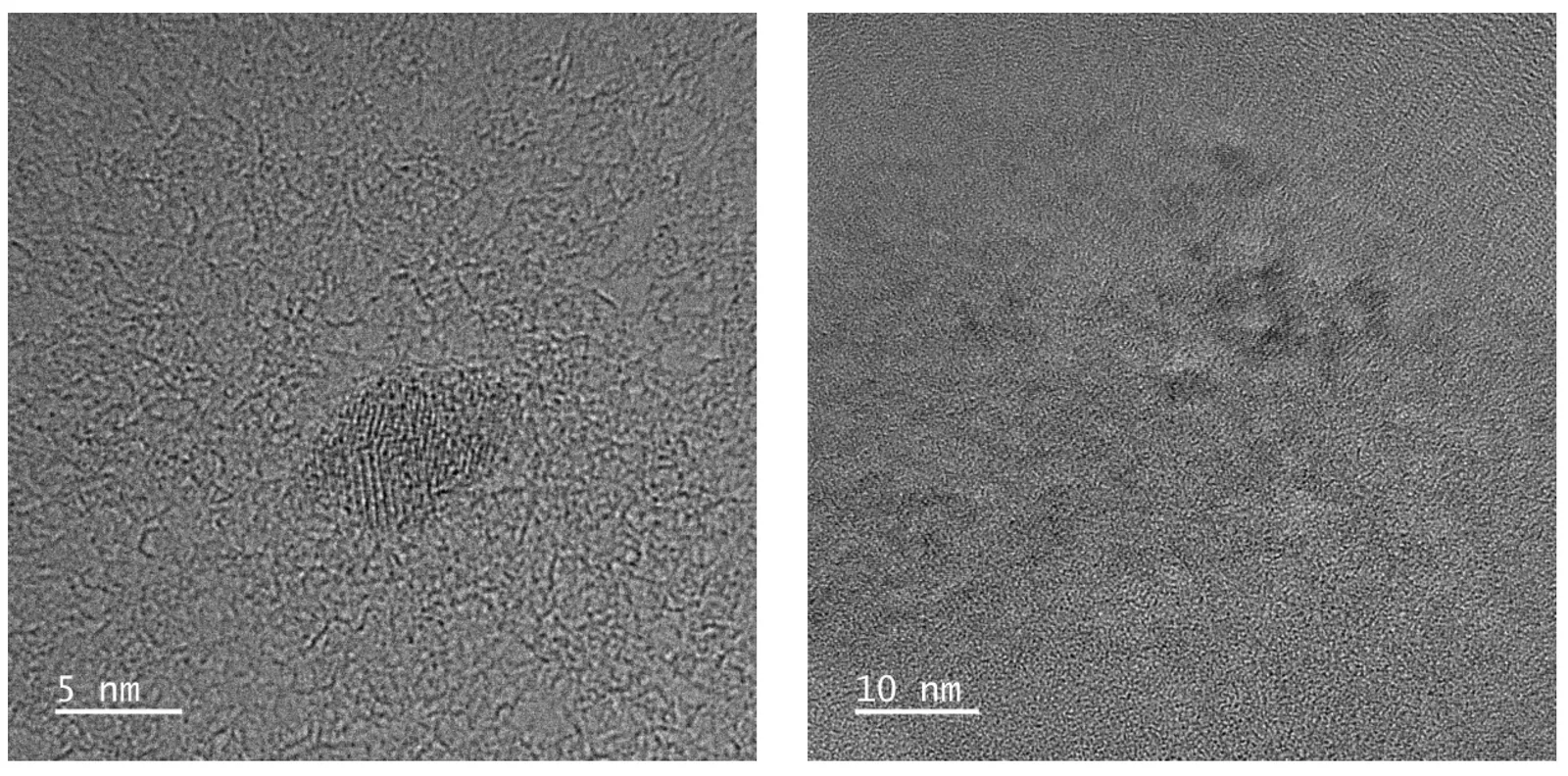
Microstructure of FNCQs.

The microstructure of FNCQs is shown in Figure 4. The morphology of the FNCQs was predominantly spherical to subspherical, along with a minor population of ellipsoidal and irregularly shaped particles. The overall dispersion was satisfactory, and no significant aggregation was detected within the sample.

**Figure 5:**
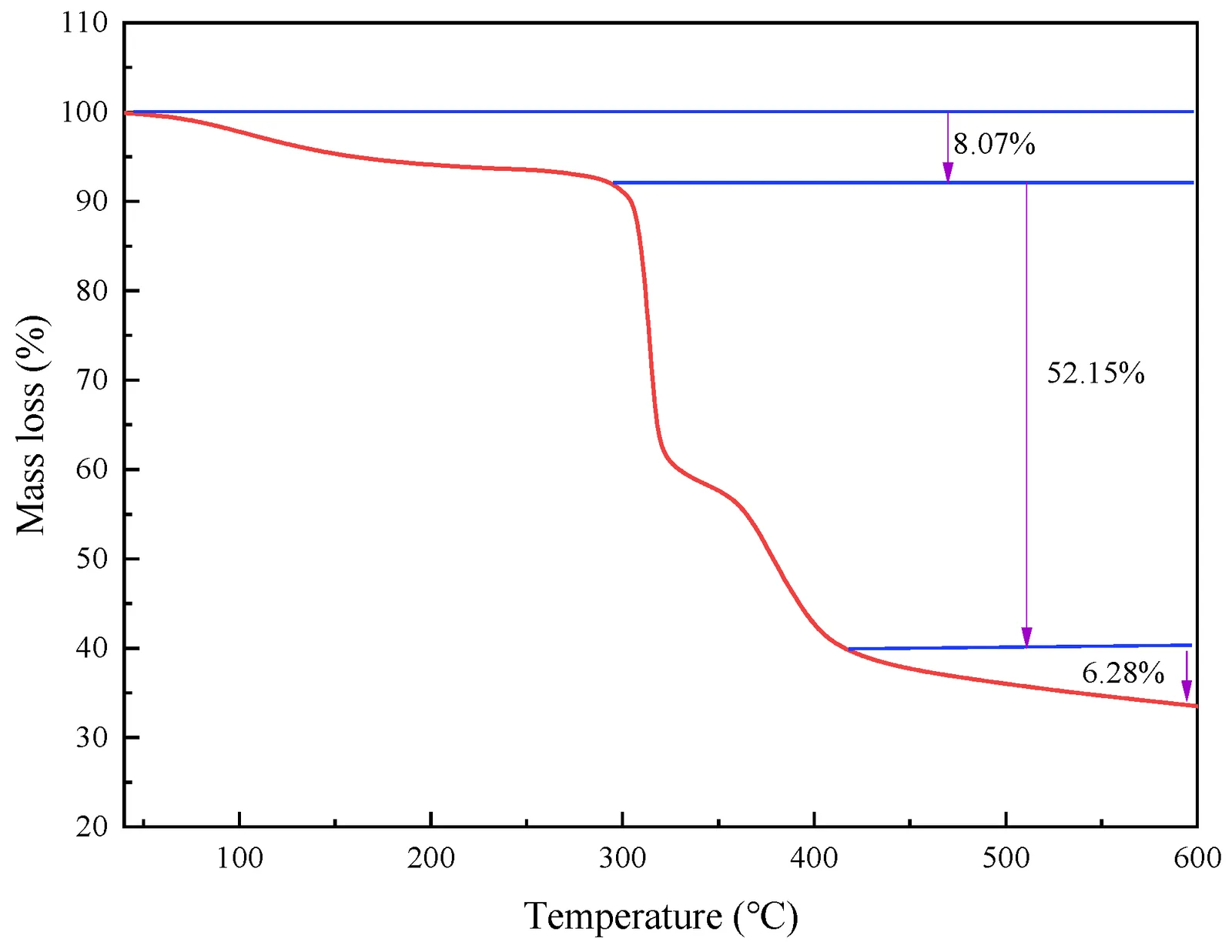
Thermal gravimetric curve of QDHSTP.

The thermal stability of QDHSTP was assessed by thermogravimetric analysis (TGA), and the corresponding weight-loss curve is provided in Figure 5. As shown in the TGA profile, the thermal degradation of QDHSTP proceeded through three separate stages. The initial stage (40–295 °C) involved an 8.07% mass decrease, associated with the loss of physically adsorbed and structural water. The intermediate stage (295–418 °C) featured the most pronounced mass drop (52.15%), stemming from amide group imidation and the thermal decomposition of hydrophobic moieties. The final stage (above 418 °C) yielded a minor mass loss of 6.28%, which likely reflects the carbonization of the polymer structure. The TGA data thus confirm that QDHSTP has satisfactory thermal resistance.

**Figure 6:**
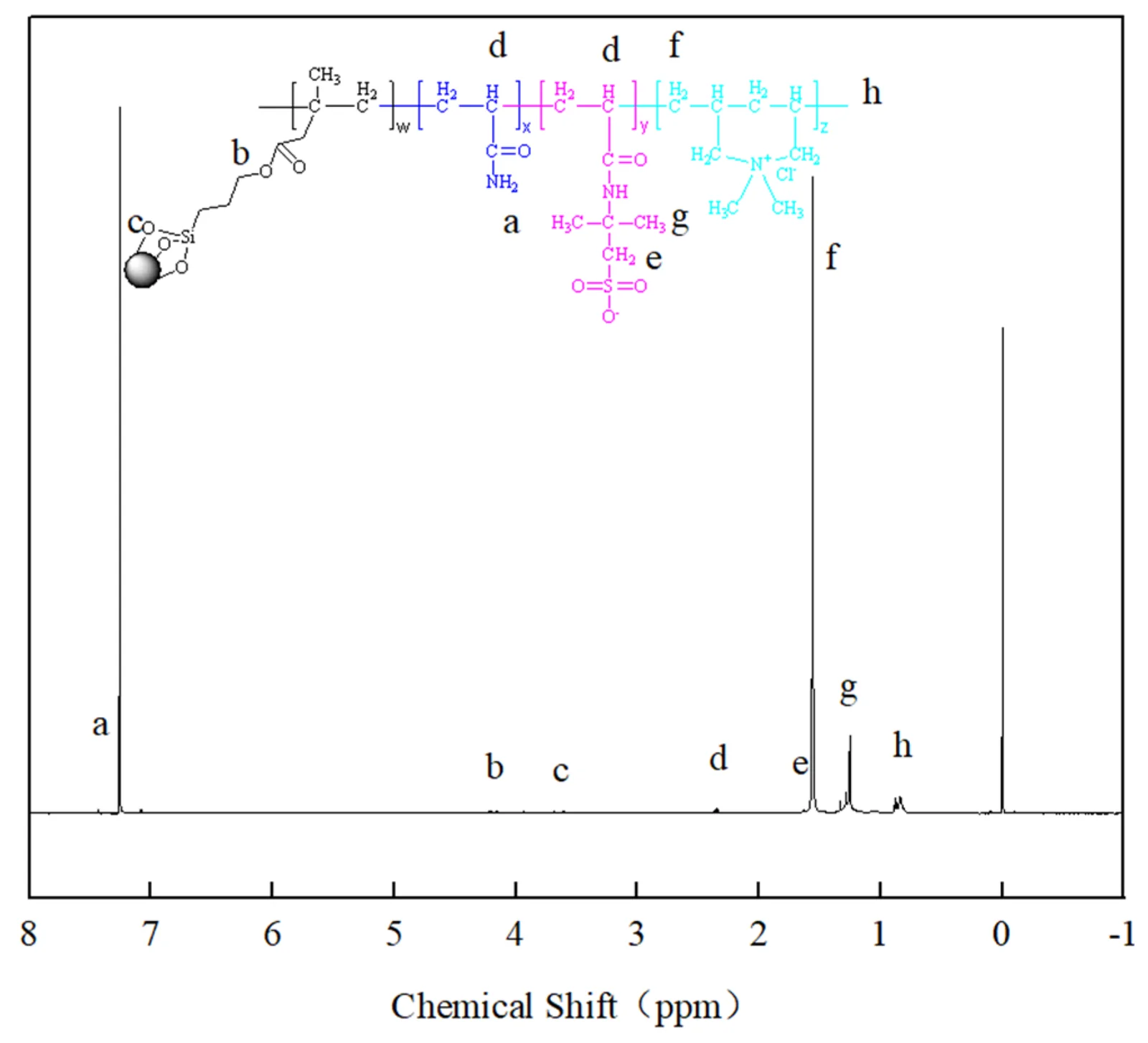
^1^H NMR spectra of QDHSTP.

The ^1^H NMR spectrum of the QDHSTP sample is shown in Figure 6, with the corresponding chemical shifts and assignments as follows: 7.30 (a, 2H) is assigned to the amide proton (-CONH_2_) of the AM unit; 4.10 (b, 1H) is assigned to the hydroxy methine (-CH(OH)-) in NCQD; 3.60 (c, 3H) is assigned to the methoxy group (-OCH_3_) from the NCQD modifier; 2.30 (d, 1H) is assigned to the methine proton (-CH-) adjacent to the carbonyl group on the polymer backbone; 1.60 (e, 2H) is assigned to the methylene group (-CH_2_-SO_3_H) directly connected to the sulfonic acid group in AMPS; 1.50 (f, 2H) is assigned to the methylene protons (-CH_2_-) on the QDHSTP backbone; 1.25 (g, 6H) is assigned to the two methyl groups (-C(CH_3_)_2_-) in the AMPS unit; and 0.80 (h, 3H) is assigned to the terminal methyl group (-CH_3_) at the QDHSTP chain end. The obtained data provide clear evidence that the synthesized polymer corresponds to QDHST.

**Figure 7:**
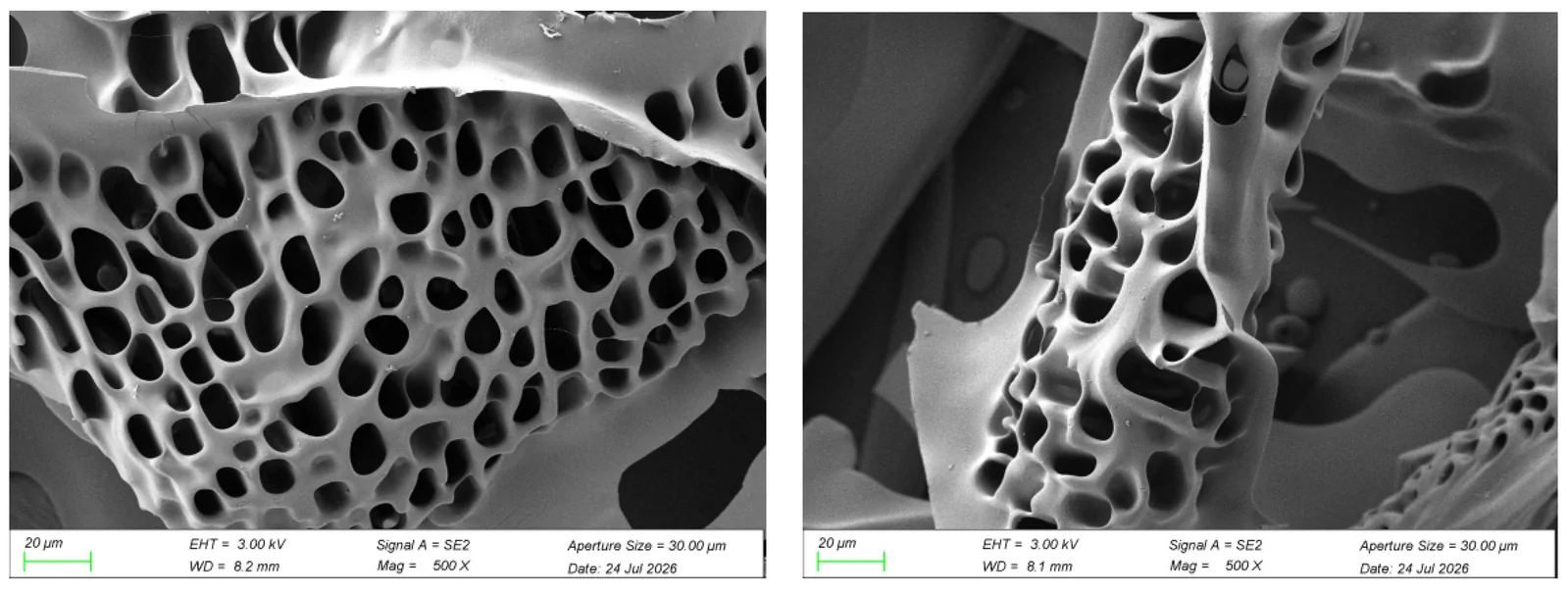
The SEM images of QDHSTP.

Scanning electron microscopy (SEM) has been widely employed to resolve the microstructural topology of polymer chains. SEM micrographs at 500× magnification (Figure 7) were captured from distinct regions of the same dried polymer specimen. The continuous three-dimensional porous network observed in the SEM micrographs is constructed by the QDHSTP macromolecular backbone, a terpolymer of AM, AMPS, and DMDAAC. In this architecture, the AM units provide abundant hydrophilic amide groups that ensure chain hydration and expansion, establishing the fundamental pore framework. The AMPS units introduce strong negative charges from sulfonate groups, and the resulting intermolecular electrostatic repulsion further stretches the polymer chains, enlarging the pore dimensions. Meanwhile, the DMDAAC cationic units balance the system charge, alleviating chain coiling induced by salt ions. Importantly, the covalently grafted FNCQDs act as physical crosslinking nodes that reinforce intermolecular entanglements, effectively stabilizing the porous structure and preventing network collapse during drying. Although minor morphological variations (e.g., oriented/wrinkled domains) were observed across different sample regions, these are attributed to non-uniform mechanical stress during freeze-drying preparation rather than intrinsic heterogeneity of the polymer network. The overall interconnected integrity remains consistent, confirming the robust structural basis for viscosity enhancement.

It should be noted that both SEM images presented in this study were obtained from the same dried QDHSTP specimen, and the intrinsic entangled network of the polymer chains remains identical throughout the sample. The pore framework is uniformly constructed by the AM/AMPS/DMDAAC/FNCQDs copolymer backbone, with the FNCQDs serving as covalent crosslinking nodes that reinforce molecular entanglements. The local morphological variations between regions—namely, the coexistence of isotropic rounded pores and wrinkled oriented structures—are attributed exclusively to non-uniform, drying-induced stress during sample preparation. In regions with faster solvent evaporation, the polymer chains undergo constrained contraction, yielding oriented and wrinkled pore channels; conversely, in regions with slower evaporation, the chains relax more fully, forming uniform and isotropic pore structures. These differences are thus artifacts of the freeze-drying process rather than indicators of intrinsic heterogeneity in the polymer network. The overall interconnected network integrity remains preserved throughout the sample, ensuring stable thickening performance regardless of local morphological details. The uninterrupted entanglement network serves as the fundamental mechanism for viscosity enhancement. In aqueous solution, water molecules are trapped within the interstitial voids of the entangled chains, significantly reducing free water mobility and elevating apparent viscosity.

### 3.2. Water Solubility

**Figure 8:**
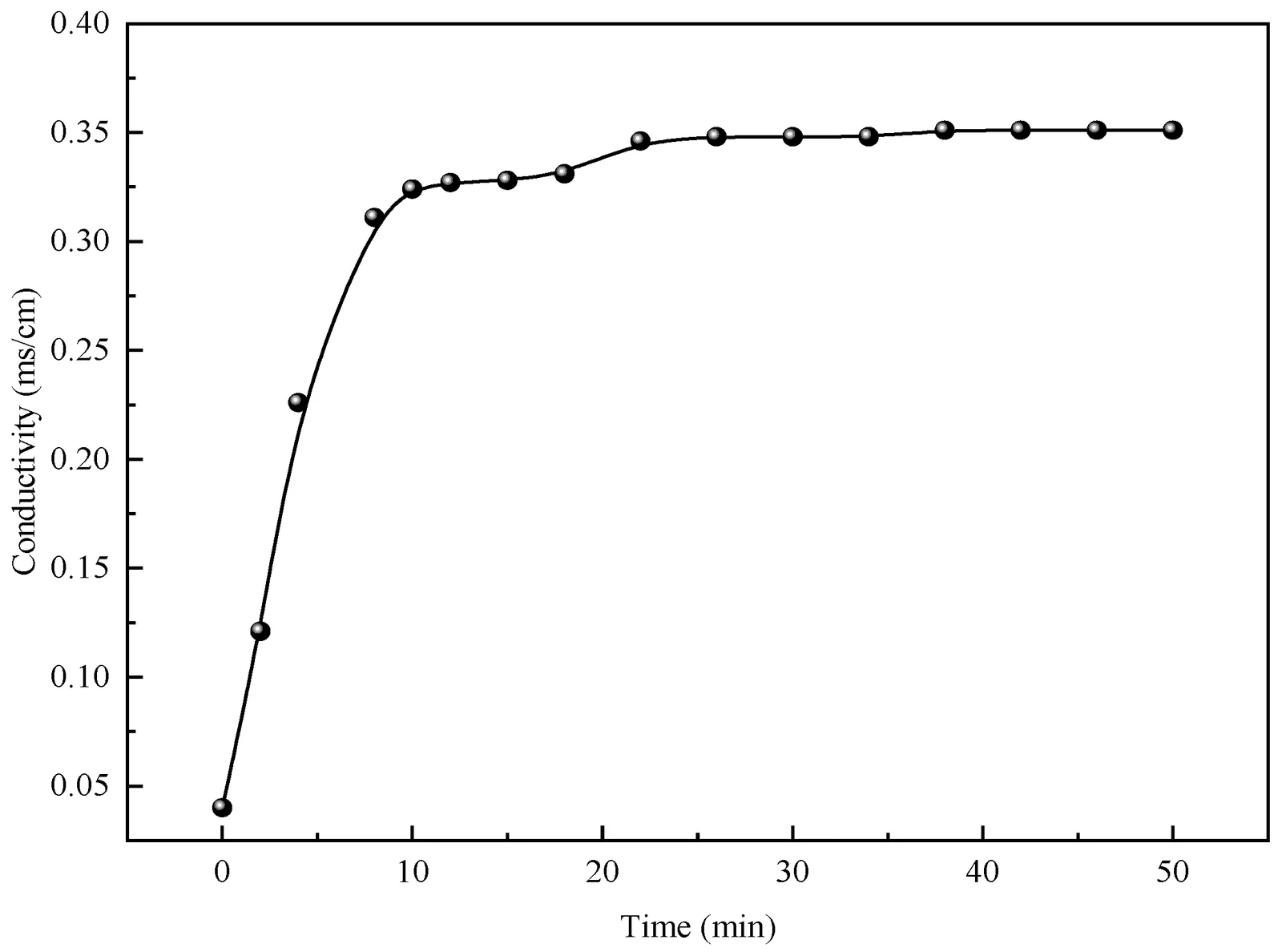
Curves of conductivity changes in the dissolution process of the QDHSTP.

One concern associated with the introduction of FNCQDs is the possible deterioration of the water solubility of QDHSTP, which may compromise its field performance. To clarify this issue, the dissolution process of QDHSTP was monitored by recording the solution conductivity as a function of time at a concentration of 2000 mg/L (Figure 8). During the initial period, a steady rise in conductivity was observed, attributable to the progressive release of polymer molecules into the aqueous medium. This was followed by a stable plateau, signifying that the polymer had reached complete dissolution. The rapid hydration of QDHSTP, as evidenced by the quick leveling of conductivity, confirms that its water solubility is fully adequate for field deployment.

### 3.3. Thickening Ability

**Figure 9:**
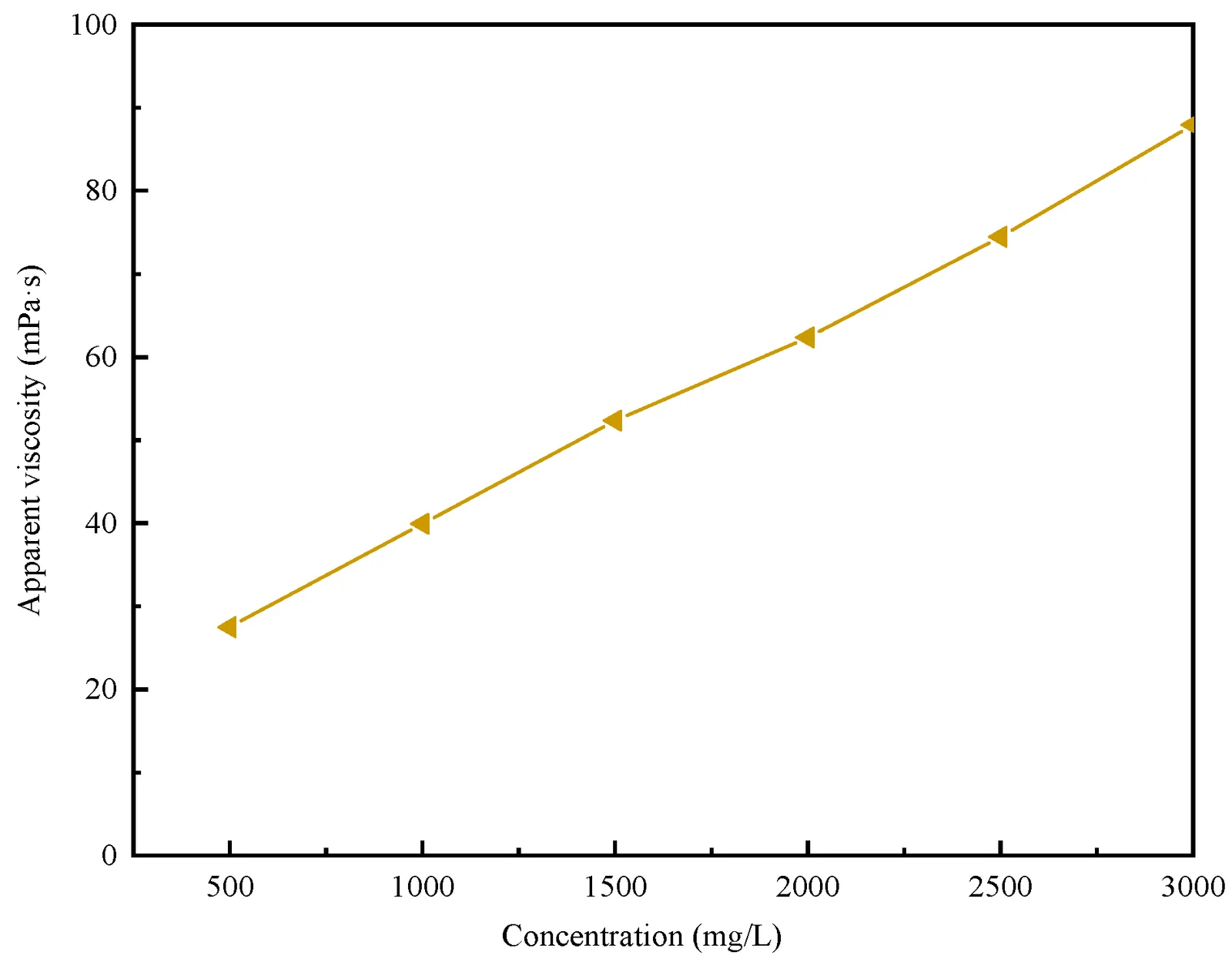
Relationship between apparent viscosity and concentration.

Viscosity enhancement is a key functional attribute of polymer solutions used in chemical flooding, as it directly influences the mobility ratio and the ultimate sweep of the reservoir. To characterize this property, QDHSTP and HSTP were dissolved in deionized water at various concentrations, and the resulting solutions were tested at 30 °C and a shear rate of 7.34 s^−1^. The viscosity-concentration relationship, illustrated in Figure 9, follows a linear trend without any anomalous discontinuities, demonstrating that no critical aggregation threshold exists within the tested range. This linear response is interpreted as a consequence of electrostatic repulsion between like-charged moieties on the polymer, which maintains the chains in an extended conformation and thereby maximizes their hydrodynamic size. Compared with HSTP, QDHSTP exhibited better thickening ability (the apparent viscosity of QDHSTP is about 15% greater than that of HSTP at equivalent concentration); the main reasons are as follows: (1) The abundant hydrophilic groups (such as –OH and –NH_2_) on the FNCQDs surface enhance the overall hydrophilicity of the polymer, promoting the formation of a thick hydration layer around the polymer chains. This increases the hydrodynamic volume and thus improves the thickening effect. (2) The covalently grafted FNCQDs act as physical crosslinking nodes, strengthening ordered intermolecular chain entanglements and forming a more compact three-dimensional network structure. This network effectively traps free water, increasing internal frictional resistance and apparent viscosity. (3) The positive charges on the FNCQDs surface, in synergy with the negative charges from the AMPS sulfonate groups, enhance intramolecular and intermolecular electrostatic repulsion, allowing the polymer chains to adopt a fully extended conformation in pure water. These three synergistic mechanisms collectively endow QDHSTP with excellent thickening performance and a linear concentration-dependent viscosity behavior.

### 3.4. Salt Tolerance of QDHSTP

**Figure 10:**
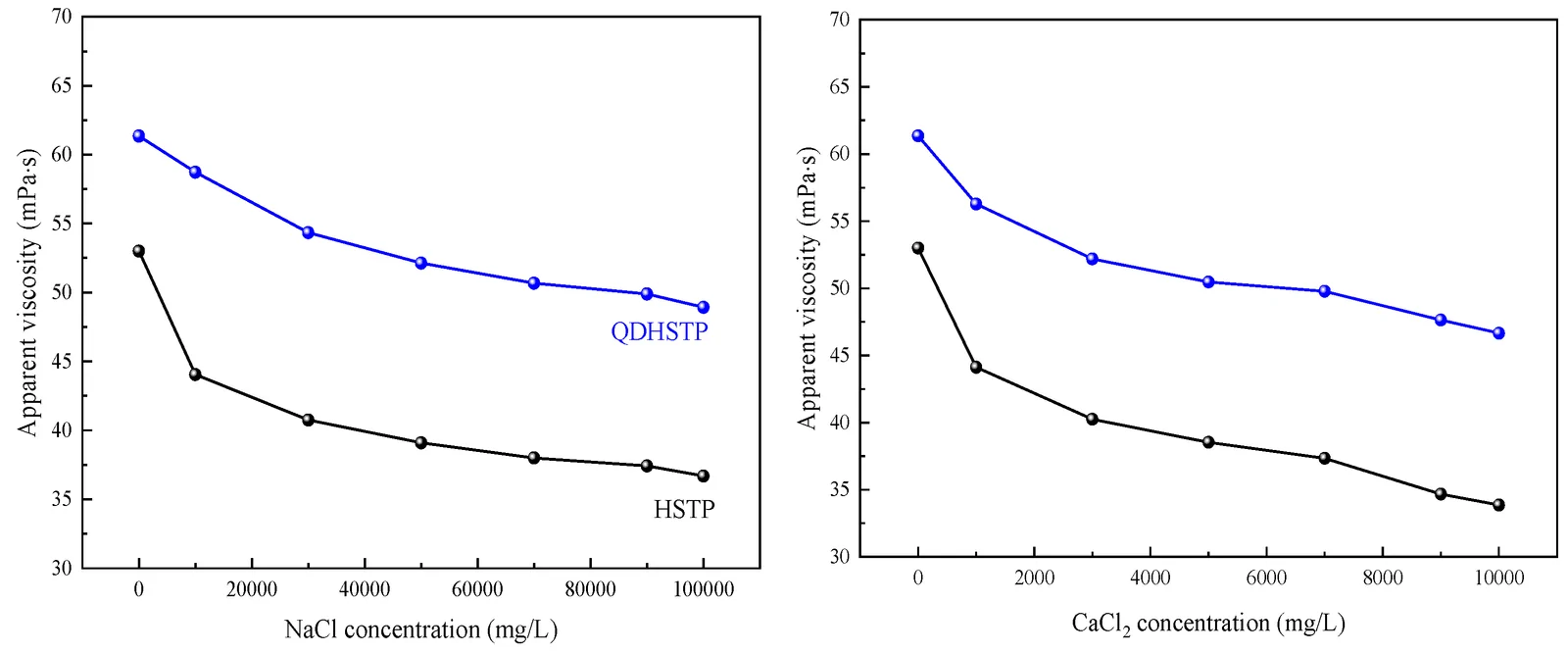
Effect of salt concentration on apparent viscosity (QDHSTP concentration, 0.2 wt%).

The salt tolerance of QDHSTP and HSTP were evaluated by monitoring the viscosity change of a 0.2 wt% QDHSTP and HSTP solution with increasing concentrations of NaCl and CaCl_2_ at 30 °C and a shear rate of 7.34 s^−1^. The results showed a progressive decline in viscosity with rising salinity (Figure 10). For NaCl, a viscosity retention of 95.71% was achieved at 1 × 10^4^ mg/L (QDHSTP), while further elevation of salinity to 1 × 10^4^ mg/L reduced the viscosity to 48.92 mPa·s, corresponding to a retention of 79.74%. For CaCl_2_, the retention was 91.74% at 1 × 10^3^ mg/L (QDHSTP), which decreased to 46.66 mPa·s (retention 76.06%) at 1 × 10^4^ mg/L. Compared with HSTP, the performance of QDHSTP is significantly improved. The main reason is that FNCQDs enhance the salt resistance of QDHSTP through three synergistic mechanisms: (1) The positive charges on FNCQDs surfaces generate a local electric field around the polymer chains, repelling Na^+^/Ca^2+^ cations and weakening their shielding and compression effects on the sulfonate groups, thereby maintaining the extended chain conformation. (2) The oxygen-containing functional groups (such as –OH and –C=O) on FNCQDs surfaces preferentially coordinate with Ca^2+^/Mg^2+^, reducing the direct interaction between divalent ions and sulfonate groups and inhibiting crosslinking precipitation. (3) The covalently grafted rigid FNCQDs hinder the hydrophobic aggregation of polymer chains compressed by salts, preserving the integrity of the three-dimensional network.

### 3.5. Temperature Resistance of QDHSTP

**Figure 11:**
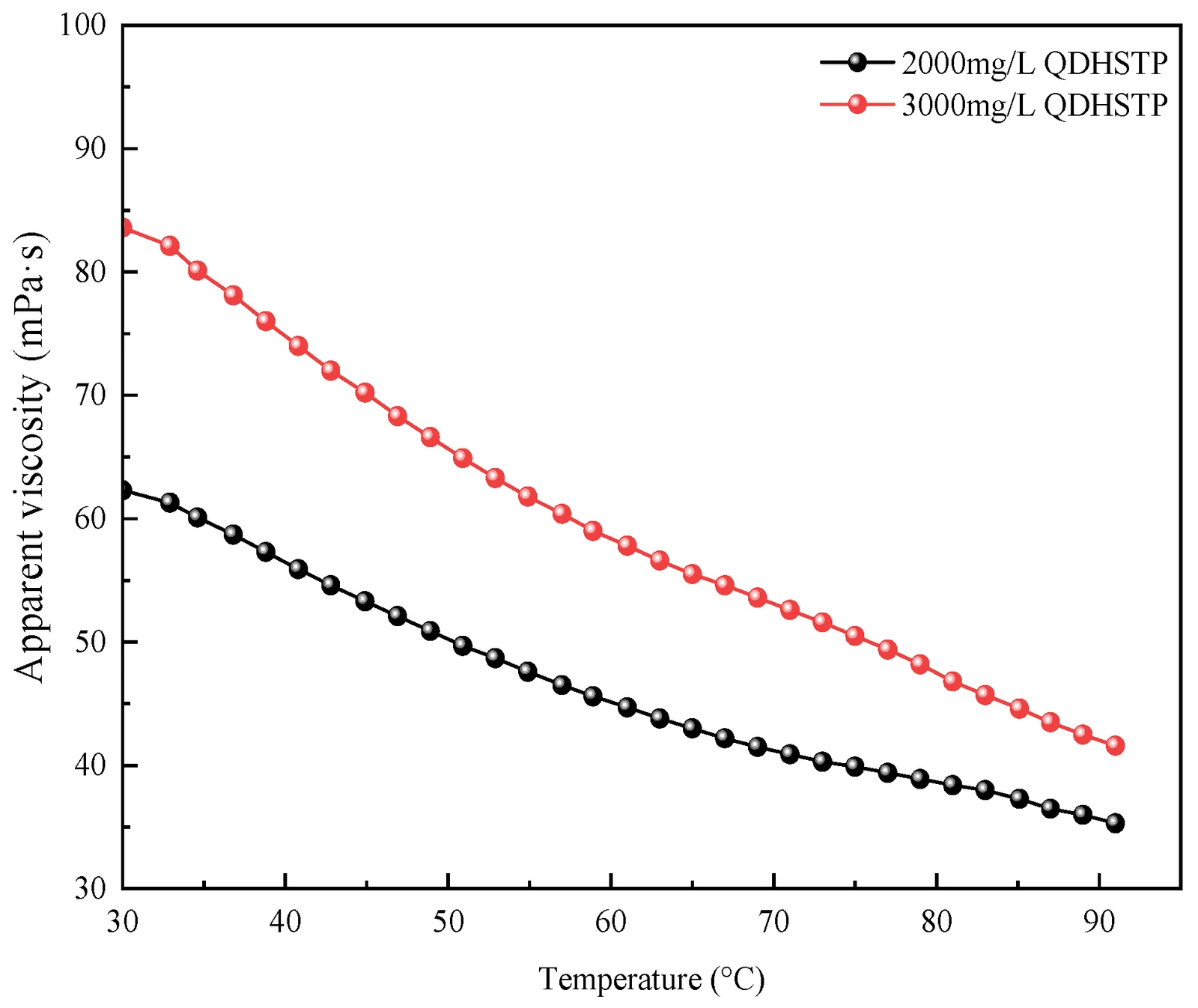
Effect of temperature on apparent viscosity of QDHSTP solution.

The viscosity of the QDHSTP solution (0.2 wt%) was measured at a shear rate of 7.34 s^−1^ as a function of temperature (Figure 11). A monotonic decrease in viscosity was observed with increasing temperature, and a 43.3–50.2% reduction was recorded at 90 °C. This viscosity loss can be mainly attributed to the enhanced thermal motion of polymer chains at elevated temperatures, which weakens both the intermolecular interactions and the electrostatic forces. Such changes are detrimental to the maintenance of the three-dimensional network structure of QDHSTP, and consequently reduce the frictional resistance between molecular chains, resulting in a notable drop in viscosity.

### 3.6. Rheological Properties of QDHSTP

**Figure 12:**
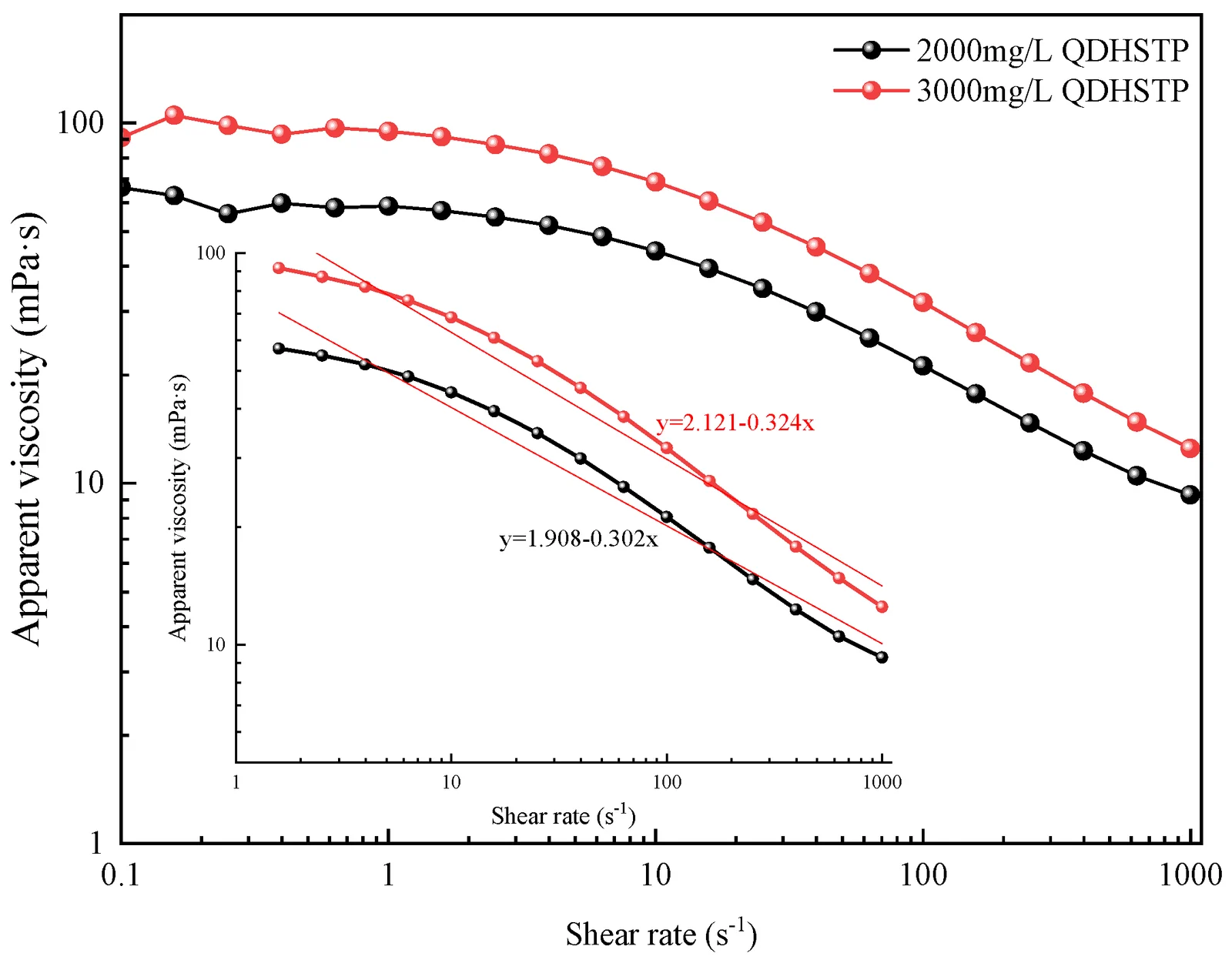
The rheological properties of QDHSTP at different concentrations.

The relationship between shear rate and apparent viscosity is shown in Figure 12. When the shear rate is below 1 s^−1^, the motion of QDHSTP molecular chains is dominated by thermal Brownian motion. The external shear force is insufficient to stretch or disentangle the chain entanglements or hydrogen bonds. The QDHSTP chains remain in their relaxed conformation, and the molecular entanglement network remains intact and stable. The internal frictional resistance of the fluid does not change significantly with slight variations in shear rate. The orientational stretching effect induced by shear can be completely counteracted by the random thermal motion of the molecules, and there is no obvious change in molecular arrangement or network structure. Consequently, the solution viscosity remains essentially constant, which is consistent with the rheological characteristics of the polymeric Newtonian plateau (zero-shear plateau).

When the shear rate exceeds 1 s^−1^, the shear force overcomes the constraints imposed by molecular thermal motion and intermolecular interactions, leading to the disruption of multiscale structures. On the one hand, the high shear rate stretches the long molecular chains along the flow direction, breaking a large number of chain entanglement nodes. The macromolecules gradually transition from random coils to an oriented and extended conformation aligned with the flow direction. The contact area between molecules decreases, resulting in a significant reduction in internal frictional resistance. On the other hand, QDHSTP relies on intermolecular hydrogen bonds and electrostatic interactions to construct a three-dimensional thickening network. Under high shear, the aggregates are disrupted, the network is damaged, and it can no longer maintain the high-viscosity structure.

When the shear rate exceeds 1 s^−1^, the relationship between the apparent viscosity and the shear rate for the non-Newtonian polymer solution was fitted using the power-law model, the mathematical form of which is provided in Equation (4).

(4) $$\eta \;=\;k\;\gamma^{n-1}$$

The variables *η*, *k*, *γ*, and n correspond to the viscosity, consistency factor, shear rate, and flow behavior index, respectively. The flow behavior index n is a measure of the shear-thinning character of the fluid, and the consistency factor *k* signifies its thickening effectiveness.

As the QDHSTP concentration increased from 2000 to 3000 mg/L, the flow behavior index *n* decreased from 0.698 to 0.676, whereas the consistency coefficient *k* exhibited a marked increase from 80.91 to 131.13 mPa·s^n^, indicating an inverse correlation between these two rheological indices. This opposing trend demonstrates that elevating the QDHSTP concentration simultaneously enhances both the shear-thinning behavior and the thickening capability of the QDHSTP system.

**Figure 13:**
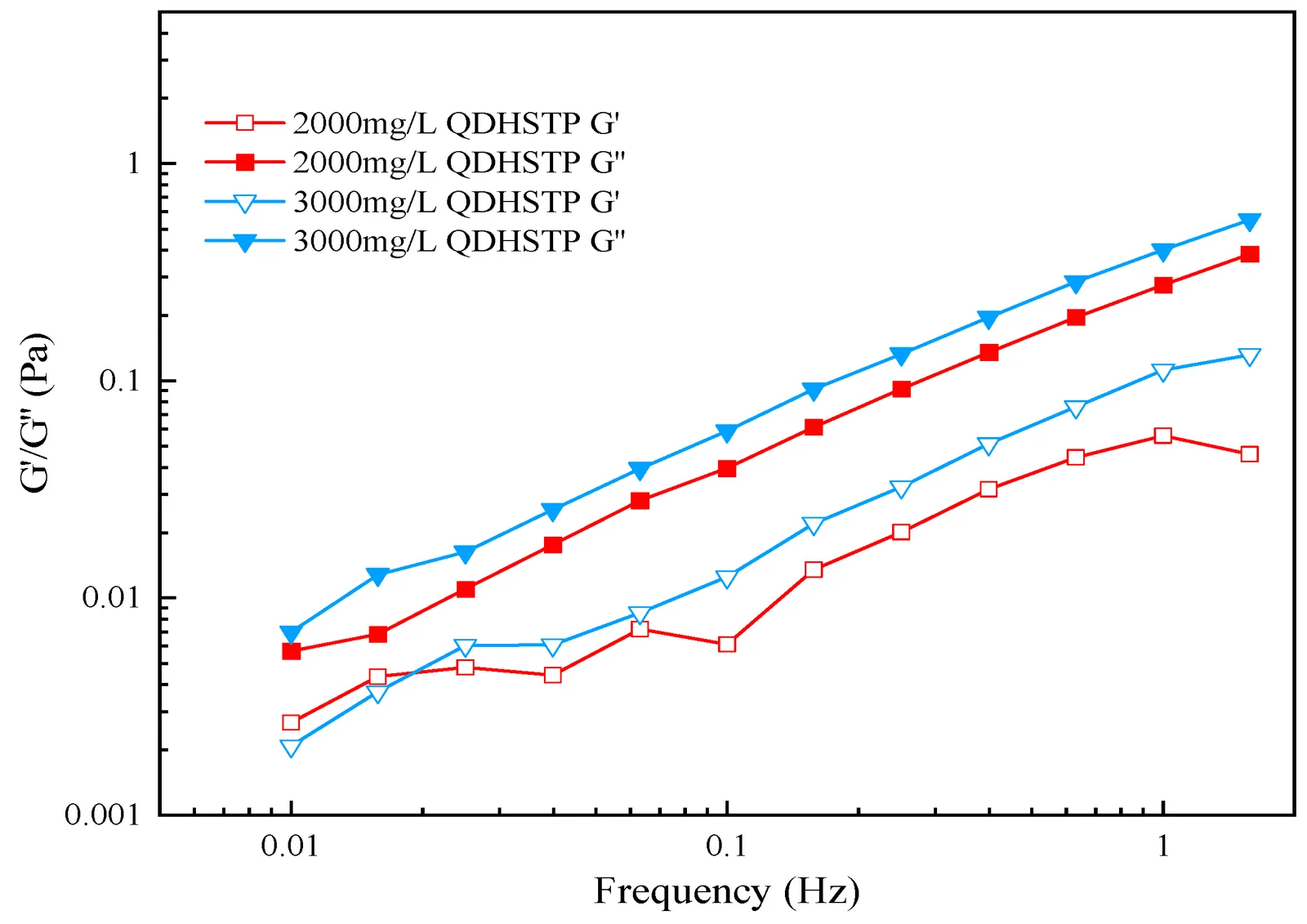
Storage (*G*′) and loss (*G*″) modulus as a function of frequency for QDHSTP solution.

In addition, the frequency-dependent evolution of the storage modulus (*G*′) and loss modulus (*G*″) is depicted in Figure 13. For both concentration systems, the *G*″ remains higher than the storage modulus across the entire test frequency range, indicating that the fluids are viscoelastic with viscosity-dominated behavior. The moduli of both systems increase with increasing oscillation frequency, and the elastic component shows a greater sensitivity to frequency variations. Increasing the polymer concentration simultaneously enhances both the viscosity and elasticity of the solution through increased molecular entanglements and intermolecular associations. For the low-concentration sample, some fluctuations in elasticity are observed at low frequencies, whereas the high-concentration system exhibits better stability of the molecular network structure. These rheological characteristics support the applicability of the polymer flooding system to adapt to both high- and low-velocity environments in reservoir formations, balancing field injection performance with in-depth profile control and plugging effectiveness.

### 3.7. Enhanced Oil Recovery Potential

Table 3 lists the average pore radium (r, µm) of core sample for brine (calculated according to Equation (4)), and the residual resistance factor (fr) and permeability reduction factor (frr) determined from QDHSTP flooding experiments.

*r*^2^ = (8·k/*φ*) (5)

where k is the permeability for brine, mD; and *φ* is porosity.

The average pore-throat size of the artificial core is 11.46 µm, which is significantly larger than the estimated hydrodynamic radius of QDHSTP chains. This size disparity ensures unimpeded polymer injectivity while still allowing effective retention through adsorption and hydrodynamic entrapment at pore constrictions. The high fr and frr indicate that QDHSTP molecules are effectively retained within the 11.46 µm pore network, substantially reducing the permeability to subsequent water. The resulting flow diversion effect directs the displacing fluid into previously unswept low-permeability regions, while the viscoelastic relaxation of entrapped chains contributes to residual oil detachment from pore surfaces. Consequently, the combination of enhanced sweep efficiency and improved microscale displacement underlies the observed 29.8% incremental oil recovery.

**Figure 14:**
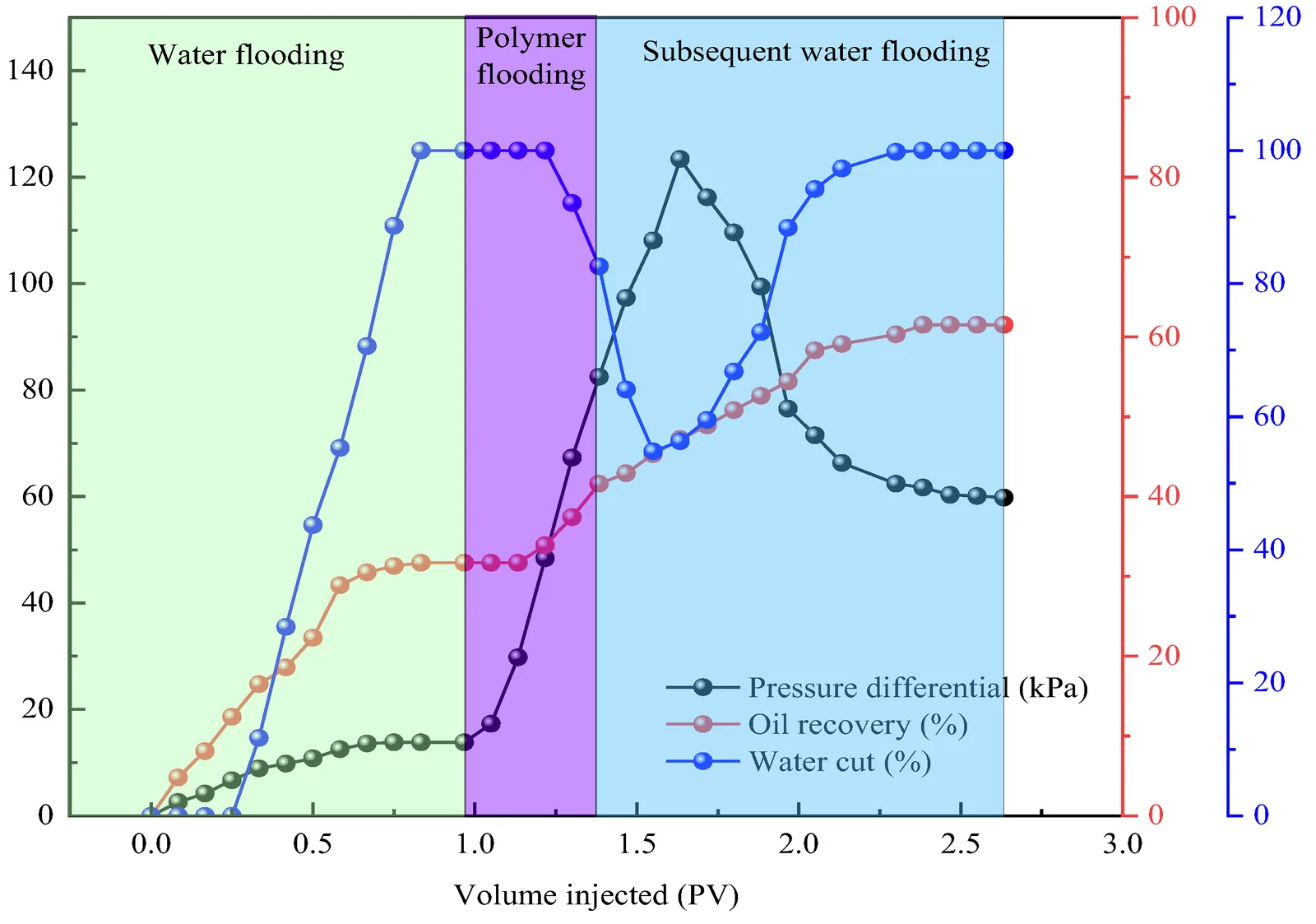
Evolution of differential pressure, water cut, and oil recovery with injection volume during the displacement process.

The dynamic evolution of differential pressure, water cut, and oil recovery throughout the displacement process is illustrated in Figure 14. In the primary water flooding stage, a steady differential pressure of 13.8 kPa was recorded, corresponding to an oil recovery factor of 31.7%. Following QDHSTP flooding and the subsequent water flooding, the differential pressure peaked at 123.4 kPa, while the water cut declined to 45.3%, ultimately yielding an incremental oil recovery of 29.8% over the water flooding baseline. Such improvements can be ascribed to the superior viscosifying ability and shear tolerance of the QDHSTP system. Notably, even after mechanical degradation caused by flow through porous media, the polymer solution retained robust mobility control performance, which contributed substantially to the enlargement of sweep volume and the overall enhancement in displacement efficiency.

## 4. Conclusions

In this study, a quantum dot-hybridized temperature- and salt-resistant polymer (QDHSTP) was successfully synthesized through thermally free-radical micellar copolymerization, with FNCQDs covalently incorporated into the polymer backbone. The following conclusions can be drawn:

(1) Structural characterization by FTIR and ^1^H NMR confirmed the successful integration of FNCQDs into the polymer backbone, with characteristic absorption bands and chemical shifts corresponding to the AM, AMPS, DMDAAC, and NCQD components. TGA analysis revealed a three-stage degradation profile with satisfactory thermal stability up to 295 °C, while SEM observations demonstrated a continuous, interconnected three-dimensional porous network with uniform chain entanglement, providing the structural basis for effective viscosity enhancement.
(2) The polymer exhibited rapid water dissolution and a linear viscosity increase with concentration, indicating favorable hydration behavior. Rheological analysis showed that QDHSTP solutions follow the power-law model with pronounced shear-thinning characteristics. Increasing polymer concentration simultaneously enhanced both shear-thinning behavior and thickening capability, as reflected by the inverse trends in *n* and *k* values, and improved the stability of the molecular network structure at low frequencies.
(3) The incorporation of quantum dots effectively mitigated salt- and temperature-induced viscosity loss. At 1 × 10^4^ mg/L NaCl and CaCl_2_, viscosity retentions reached 79.74% and 76.06%, respectively, and the polymer maintained appreciable viscosity at 90 °C. The quantum dots provided a robust spatial network and electrostatic stabilization that prevented polymer aggregation and suppressed metal-ion complexation and precipitation. Accordingly, QDHSTP possesses superior thickening performance and salt-tolerant properties compared with HSTP.
(4) Core flooding experiments demonstrated that QDHSTP achieved a 29.8% incremental oil recovery over waterflooding, with a dramatic increase in differential pressure from 13.8 to 123.4 kPa. The high resistance factor and residual resistance factor confirmed effective mobility control and favorable retention behavior within porous media, validating the practical applicability of QDHSTP for enhanced oil recovery in heterogeneous reservoirs.

Overall, the QDHSTP system offers a promising nanomaterial-enhanced polymer flooding strategy that effectively addresses the temperature and salinity limitations of conventional HPAM, providing a robust and field-deployable solution for tertiary oil recovery in challenging reservoir conditions.

### Limitations and Future Directions

Several limitations of this study should be acknowledged. The long-term thermal stability, shear resistance, and economic feasibility of QDHSTP have not been evaluated. Future work will focus on systematic aging tests, cost optimization of FNCQDs synthesis, and multi-permeability core flooding with replicated trials to validate the field applicability of this covalent nanohybrid strategy.

## Figures and Tables

**Table 1:** Basic parameters of core samples.

Core Sample	Diameter (cm)	Length (cm)	Porosity (%)	Water Permeability (mD)	Oil Saturation (%)
1000-1	3.801	7.034	22.58	487.63	/
1000-2	3.799	7.055	23.46	501.42	77.96

**Table 2:** Composition of water for water flooding.

Ion	Na^+^ + K^+^	Ca^2+^	Mg^2+^	Cl^−^	HCO_3_^−^	SO_4_^2−^	Total
Concentration (mg/L)	9258	340	96	9149	245	368	20,260

**Table 3:** *f_r_* and *f_rr_* of core sample 1000-1.

Core Sample	Porosity (%)	Water Permeability (mD)	*r* (μm)	*f_r_*	*f_rr_*
1000-1	22.58	487.63	11.46	49.32	11.57

## Data Availability

The original contributions presented in this study are included in the article. Further inquiries can be directed to the corresponding author.
